# Pneumonitis Associated with Immune Checkpoint Inhibitors and Targeted Anticancer Therapies: A Retrospective Case Series of 12 Patients

**DOI:** 10.3390/diseases14090313

**Published:** 2026-08-27

**Authors:** Claudia Lucia Toma, Ștefania Florina Oprea, Ștefan Dumitrache-Rujinski, Ionela Nicoleta Belaconi, Daniela Jipa-Dună, Cristian Cojocaru, Alexandra Maria Cristea, Camelia Cristina Diaconu, Dragos Cosmin Zaharia

**Affiliations:** 1 Faculty of Medicine, Carol Davila University of Medicine and Pharmacy, 050474 Bucharest, Romania; stefan.dumitrache@umfcd.ro (Ș.D.-R.); ionela.belaconi@umfcd.ro (I.N.B.); daniela.jipa@umfcd.ro (D.J.-D.); alexandra.maria.sora@gmail.com (A.M.C.); camelia.diaconu@umfcd.ro (C.C.D.); dragos.zaharia@umfcd.ro (D.C.Z.); 2 Pneumology IV Department, Marius Nasta Institute of Pulmonology, 050159 Bucharest, Romania; 3 Faculty of Medicine, Grigore T. Popa University of Medicine and Pharmacy, 700115 Iasi, Romania; cristian.cojocaru@umfiasi.ro; 4 Internal Medicine Department, Clinical Emergency Hospital of Bucharest, 105402 Bucharest, Romania

**Keywords:** immune-induced pneumonitis, target agents-induced pneumonitis, immune checkpoint inhibitor, anti-CD20-targeted agents, CDK 4/6 inhibitor

## Abstract

Background: Immunotherapy and targeted therapy have gained ground over conventional chemotherapy in treating various cancers. While pulmonary toxicity associated with these agents is rare, it represents a significant factor in both mortality and morbidity and may influence the overall success of cancer treatment. This case series report adds to the emerging evidence of cancer therapy-induced pneumonitis features and corticotherapy outcomes. Patients and methods: This single-center, retrospective case series analyzed 12 consecutive cases of patients undergoing immunotherapy (four receiving nivolumab, four receiving pembrolizumab) or targeted therapy (three receiving obinutuzumab, one receiving abemaciclib) for cancer (seven with lung cancer, three with non-Hodgkin lymphoma, one with breast cancer, one with renal cancer) who developed pneumonitis during their follow-up. Results: The interval from oncological treatment initiation to pneumonitis onset ranged from 6 to 48 months (median = 18.5), and in four patients it occurred after discontinuation of oncologic therapy. In most patients, the diagnosis was established with high probability based only on the clinical presentation, radiologic pattern, and concomitant oncologic therapy. Bronchoscopy with bronchoalveolar lavage analysis was performed in eight of the 12 patients, particularly when onset followed treatment discontinuation. The main symptom was dyspnea (10/12 cases), and three of 12 patients had respiratory failure (SpO_2_ ≤ 88%). The CTCAE severity grades were: one mild, seven moderate, three severe, and one life-threatening. The CT scan showed different patterns (7 OP, 4 NSIP-like, and 1 HP). Eleven patients received oral methylprednisolone (0.40 to 0.82 mg/kg) for 5 to 16 weeks. Two patients continued oncologic treatment, and six discontinued. Pneumonitis improved or resolved in 11 of the 12 patients; one patient deteriorated after reintroduction of immunotherapy and subsequently died from cancer-related complications. Conclusions: Immunotherapy- and targeted therapy-induced pneumonitis can express various features and severities, and prompt recognition and diagnosis based on clinical, radiologic and contextual elements are mandatory. In this small, heterogeneous series the individualized corticosteroid regimens used were followed by favorable outcomes. Our observations suggest that, in selected clinically improving patients, follow-up may rely only on clinical assessment and chest X-ray, and extensive tests may be reserved for non-responsive cases.

## 1. Introduction

In recent years, the treatment of certain types of cancer has been revolutionized by the approval of targeted therapies and immunotherapy. Although pulmonary toxicity is a rare adverse effect, the increasing use of these therapeutic agents has facilitated the recognition of such reactions.

For active immunotherapy involving immune checkpoint inhibitors (ICI), the incidence of therapy-induced pneumonitis ranges between 2.7% and 10% [1,2,3,4,5,6], with conflicting evidence that a higher incidence is observed in cases involving the combination of immunotherapeutic agents [7]. Concomitant chemotherapy seems to reduce the risk of immune-related pneumonitis [7]. In the case of molecular-targeted therapies, incidence rates vary depending on the specific agent. For instance, anti-CD20 agents such as Rituximab have been associated with a risk of inducing non-infectious pneumonitis ranging from 3.9% to 8.4% [8,9]. Conversely, the literature describes only a few reported cases of pneumonitis caused by the anti-CD20 agent obinutuzumab [10,11]. Additionally, CDK 4/6 inhibitors rarely cause pneumonitis; however, the FDA has issued warnings regarding the potential severity of these adverse events [12].

The prompt recognition of pulmonary toxicity is crucial to preventing potentially irreversible or fatal consequences. Pneumonitis is one of the leading causes of immunotherapy discontinuation [3]. Suspicion usually starts with common symptoms (progressive exertional and even at-rest dyspnea, cough, fever) in a patient with cancer undergoing targeted oncologic therapy for several months. The median time to onset is 3 months, but there is high variability (9 days to 19 months) [3], and pneumonitis can occur even several months after cessation of therapy [13]. Patients can present with mild hypoxemia (PaO_2_ more than 60 mmHg and SpO_2_ more than 88–90% but less than normal ~95%) or various degrees of respiratory failure (PaO_2_ less than 60 mmHg and SpO_2_ less than 88–90%). Clinical exam of the thorax can be normal, and lung crackles may be audible in rare cases. Postero-anterior chest X-rays can reveal a patchy interstitial pattern with mild opacity consistent mostly with ground-glass attenuation on CT scan or more intense opacity consistent with lung consolidation. Most papers that present radiologic features of oncologic targeted therapy-induced pneumonitis discuss less simple chest X-ray images and focus mainly on CT scan patterns. Inflammatory markers can have increased values but are not currently used for diagnosis. These patterns can be localized or diffuse. These clinical (symptoms, signs), contextual (cancer following targeted therapy), and paraclinical elements (chest X-ray, inflammatory markers) can suffice for a high-probability diagnosis, but most clinicians follow a more prudent approach and perform other more invasive and costly medical investigations to “confirm” the diagnosis.

HRCT scan is frequently used to characterize and localize more precisely the lung lesions as it has a higher sensitivity and specificity for interstitial lung diseases [14]; the most typical and frequent patterns are patchy ground-glass attenuation and consolidation zones distributed in both lungs which are quite similar to the aspects encountered in SARS-CoV2 pneumonia (diffuse alveolar damage/acute interstitial pneumonia/acute respiratory distress syndrome–DAD/AIP/ARDS) or organizing pneumonia (OP) [15,16]. Other patterns were also described: nonspecific interstitial pneumonia (NSIP), hypersensitivity pneumonia (HP), bronchiolitis (BL), radiation recall pneumonitis (RRP) [16], diffuse alveolar hemorrhage (DAH) or sarcoid-like reactions (SLR). Also, localized lung lesions can be more rarely encountered [10].

Bronchoscopy and lung biopsy can be performed to further characterize the existing lung injury by cell distribution patterns but often cannot distinguish other diagnoses such as various types of non-bacterial pneumonia, alveolar hemorrhage, lung congestion, non-cardiogenic pulmonary edema, sarcoidosis, lymphangitic carcinomatosis, etc. The bronchoalveolar lavage (BAL) is important to exclude an infectious cause (viral, bacterial or fungal). The bronchoalveolar lavage reveals lymphocyte-predominant cellularity most of the time [15]. Still, the existence of other patterns cannot exclude the oncologic targeted therapy lung injury, and the presence of malignant cells can be a feature of the subsidiary oncologic disease. For these considerations, these complex medical tests cannot confirm the etiologic diagnosis. Lung biopsies can also be performed, but the high variability of histopathologic lung injury patterns generated by different drugs and even within a specific class of therapeutic agents [3,15] correlated with the risks, making this approach less desirable in terms of the risk/benefit ratio.

The management of these patients is guided by symptomatology, levels of severity or presence of respiratory failure and the extent of pulmonary involvement [17] and is guided by severity grades by CTCAE criteria [18]. For asymptomatic patients, imaging surveillance alone is sufficient, without interrupting immunotherapy or targeted therapy. Depending on the severity of symptoms, discontinuation of immunotherapy or targeted therapy and the initiation of corticosteroid treatment should be considered in moderate to severe patients. If considering corticotherapy, currently there is no standard recommendation of treatment for this type of pneumonitis or of discontinuation of the incriminated therapy. Most recommendations are based on clinical experience, consensus opinion or observational studies or case series and include corticotherapy regimens of various doses (0.5–1 mg/kg) for various lengths (minimum of 6 weeks and no maximum duration recommendation) and with no standard dose tapering rhythm (at least 4 weeks, based on the initial severity, clinical response and tendency to relapse with dose decrease) [19,20].

Against this background and given that much of the existing literature focuses on immune checkpoint inhibitors and on computed tomography findings, we report a single-center, retrospective series of 12 patients who developed pneumonitis during or after treatment with immune checkpoint inhibitors or targeted anticancer agents. This series provides information on pneumonitis induced by less commonly reported anticancer agents, such as obinutuzumab and abemaciclib. It also includes patients who developed pneumonitis long after the initiation of oncologic treatment, emphasizing that drug-induced pneumonitis may occur both during treatment and after its completion. In addition, it offers radiological images of the different findings and patterns, both at diagnosis and during follow-up. Finally, it suggests evaluation by chest radiography for follow-up in selected, clinically improving patients, in order to reduce costs and avoid unnecessary, expensive investigations. However, given the small and heterogeneous group of patients and the retrospective nature of the case series, we consider these observations to be descriptive and hypothesis-generating.

## 2. Materials and Methods

### 2.1. Selection and Description of Participants

This case series includes twelve consecutive cases of pneumonitis induced by immunotherapy or targeted therapy diagnosed and managed by the authors at the Marius Nasta Institute of Pulmonology in Bucharest, Romania, between January 2022 and June 2025. The medical data of these cases were collected based on the personal data collection consent form signed by the patients on admission to the hospital, which also covers research purposes. Besides this, verbal consent for the purpose of publishing anonymized medical data was obtained from the subjects who were alive at the moment of the decision to start this study. A formal request was sent to the ethics committee of the Marius Nasta Institute of Pulmonology on 11 July 2025. It was approved by the local ethics committee of the Marius Nasta Institute of Pulmonology in August 2025 (reference number 16362/19 August 2025) and was conducted in accordance with the Declaration of Helsinki. The manuscript was reviewed by all team members.

The medical data used in this study are available and can be provided by the corresponding author upon request.

The study aims to add more information to the emerging evidence of diagnostic features and management outcomes of these cases.

### 2.2. Data Collection and Measurements

A retrospective search was performed to identify all consecutive patients admitted to the Pneumology IV Department of the Marius Nasta Institute of Pulmonology between January 2022 and June 2025, diagnosed and managed by the authors, in whom a diagnosis of oncologic therapy-induced pneumonitis was established according to the criteria described below. As no specific diagnostic code exists for oncologic therapy-induced lung disease, cases were identified by searching the discharge summaries for the keywords “drug-induced pneumonitis”, “induced pneumonitis”, “associated pneumonitis”, “immunotherapy”, “immunotherapy-induced pneumonitis”, “targeted therapy” (translated from Romanian), complemented by the treating physicians’ direct knowledge of the patients managed in the department during the study period.

Patients were excluded if the available clinical or imaging data were insufficient to support the diagnosis, or if they were lost to follow-up and did not attend their scheduled control visits, precluding assessment of the disease course.

**Figure 1:**
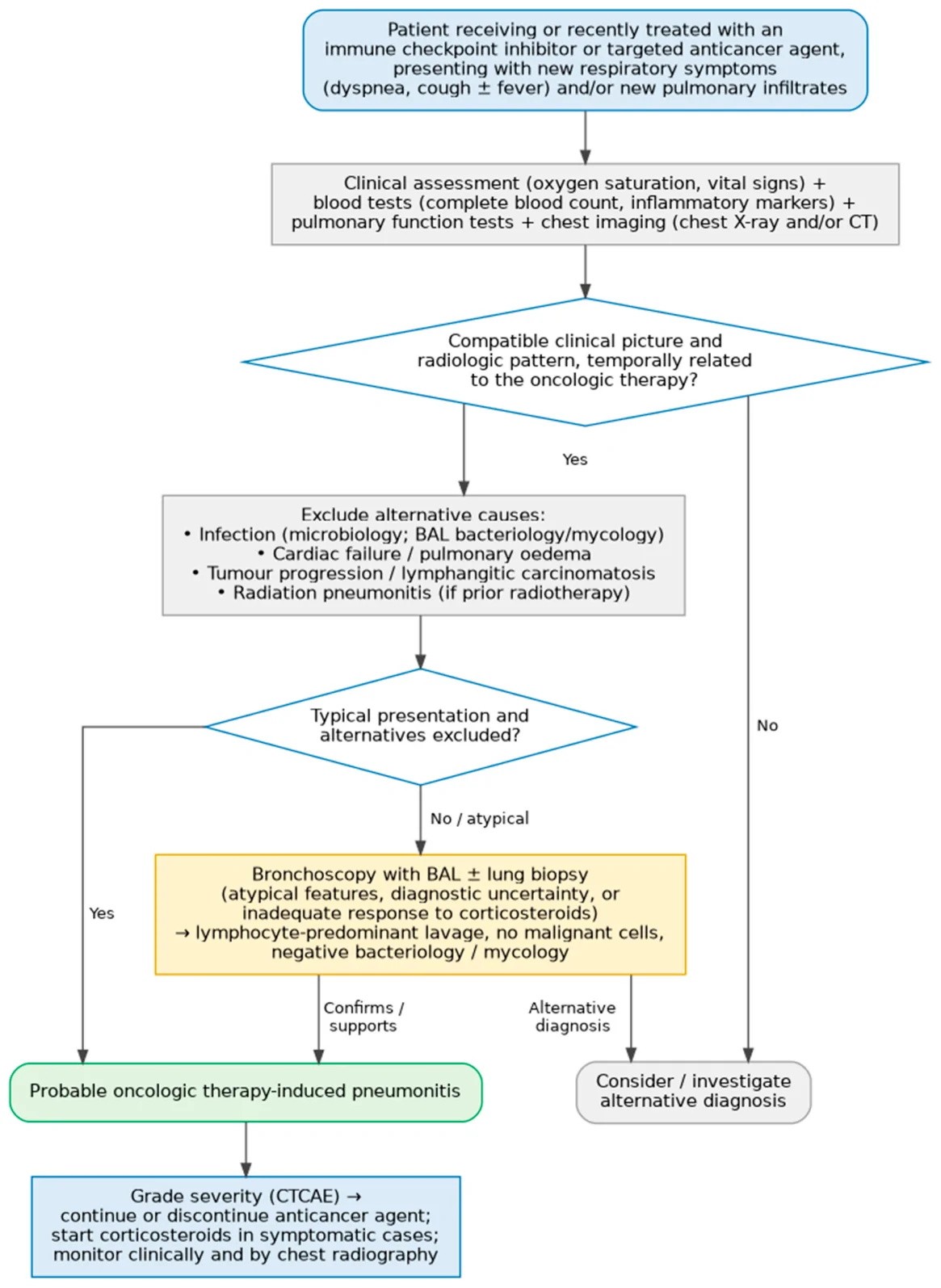
Diagnosis flowchart of oncologic therapy-induced pneumonitis. CT = computed tomograpy, BAL = bronchoalveolar lavage, CTCAE = Common Terminology Criteria for Adverse Events.

In our cohort, the diagnosis of oncologic therapy-induced pneumonitis was based on a stepwise, predominantly non-invasive approach, with bronchoscopy and biopsy reserved for selected cases (Figure 1). Pneumonitis was suspected in patients receiving, or recently treated with, an immune checkpoint inhibitor or a targeted anticancer agent who developed new respiratory symptoms (progressive dyspnea, cough, with or without fever) and/or new pulmonary infiltrates.

All patients underwent clinical evaluation, including measurement of oxygen saturation, blood pressure and heart rate, blood tests (complete blood count and inflammatory markers, namely C-reactive protein, erythrocyte sedimentation rate and fibrinogen), pulmonary function tests and chest imaging (chest radiography and/or computed tomography).

A probable diagnosis was considered when the clinical presentation and the radiologic pattern were compatible with drug-induced pneumonitis and were temporally related to the oncologic therapy. Alternative causes were actively excluded, namely pulmonary infection, cardiac failure and pulmonary edema, and lymphangitic carcinomatosis or tumor progression. Concerning radiation pneumonitis, only two patients had received radiotherapy as part of their oncologic treatment, and their radiological changes were not compatible with this type of parenchymal involvement.

Bronchoscopy with bronchoalveolar lavage was reserved for patients with atypical clinical or radiologic features, diagnostic uncertainty, or an inadequate response to corticosteroid therapy. In the 8 patients who underwent bronchoalveolar lavage, no malignant cells were identified, and bacteriological and mycological examinations were negative, with lymphocyte-predominant cellularity arguing against infection and malignant involvement. Once a probable diagnosis was established, severity was graded according to the CTCAE (Common Terminology Criteria for Adverse Events) criteria, and management—continuation or discontinuation of the anticancer agent, initiation of corticosteroids, and follow-up by clinical and radiographic assessment—was decided accordingly.

The main characteristics of the patients included in the case series are detailed in Table 1.

Each clinical case was studied in terms of history, clinical picture, basic tests (chest X-rays, blood tests), further tests (CT scans, bronchoscopy with BAL), treatment and outcome.

Imaging studies (chest X-rays and CT scans) were downloaded from the hospital’s database, anonymized, and further studied with the open-source cross-platform imaging software “Weasis–uptodate version.” A descriptive assessment of the initial CT scan was performed using the suggested features of CT patterns previously identified in the literature [16]. Based on the symptomatology, SaO_2_, and extent of lung involvement, the CTCAE version 5.0 criteria were used to grade pneumonitis severity at the initial diagnosis (Table 2) [17]. A subjective, semi-quantitative comparison of lung involvement was performed for the imaging exams obtained for each patient at the time of diagnosis/start of treatment and after a time interval (during or at the end of treatment) to assess the outcome in imaging terms. Comparative assessments were made preferably between same-type images (chest X-rays or CT scans) based on availability. These features were added to the follow-up clinical assessment (improvement or worsening of symptoms) to establish the outcome (improved, stationary, worsened, death). Improvement was defined as a clinical response with amelioration of respiratory symptoms together with regression of the radiological abnormalities; resolution was defined as complete or near-complete clearing of the pneumonitis-related radiological abnormalities. Stationary disease was defined as the absence of significant clinical or radiological change compared with the initial assessment. Worsening was defined as aggravation of pre-existing respiratory symptoms or the appearance of new ones, with or without radiological progression. In cases of discordance between clinical and radiological evolution, patients were classified according to the clinical course.

The essential collected data were centralized in tables of results, and general observations and considerations were issued by the research team. No statistical analysis could be performed on this type of data.

## 3. Results

### 3.1. Onset, Symptoms and Severity

The average and median onset durations were 19.4 and 18.5 months, respectively, ranging from 6 to 48 months. One case had an exceptionally long time to onset of 48 months. If we analyzed by drug class, the onset interval differs between groups. In the eight patients treated with ICI, the mean and median onset were 18.8 and 14 months, respectively. In the three patients treated with obinutuzumab (anti-CD20), the median onset was 24 months, with a mean of 32 months, and included the patient with the longest interval of the series (case 4, 48 months). In the only patient treated with abemaciclib (CDK4/6 inhibitor), pneumonitis occurred 19 months after treatment initiation.

The most frequent symptom observed in 10 out of 12 patients was progressive dyspnea, while only two patients reported dry cough as the sole complaint. In addition to dyspnea, four patients also presented with cough. Fever was present in a single patient (case 8), and two patients reported loss of appetite (case 3) or chest pain (case 4). Three patients presented with respiratory failure (SpO_2_ ≤ 88%) (Table 3).

Oxygen saturation at diagnosis was available for all 12 patients (median 97%, range 70–99%), and three patients (cases 8, 11 and 12) met the criterion for respiratory failure (SpO_2_ ≤ 88%). Lung function testing was feasible only in part of the series. DLCO was obtained in eight of 12 patients (median 44.6%, range 17.4–68% of predicted) and was below 80% of predicted in all of them, with a mild reduction (60–79%) in two cases, a moderate reduction (40–59%) in two cases and a severe reduction (<40%) in four cases. FVC was obtained in eight of 12 patients (median 66.3%, range 46.2–84.7% of predicted) and was below 80% of predicted in seven of them, consistent with a predominantly restrictive ventilatory pattern.

When analyzed by drug class, it seems that symptoms and severity also differed. All the patients treated with immune checkpoint inhibitors presented with dyspnea, and three of them reported cough. This group included all cases of grade 3 and 4 and all three patients with respiratory failure. In contrast, the three patients treated with obinutuzumab had a milder presentation, graded 2. The single patient treated with abemaciclib presented with dry cough as the only complaint, was graded 1, and had a normal oxygen saturation (99%). These differences are descriptive and, given the very small size of the non-checkpoint inhibitor groups and also the different types of cancer (pulmonary vs. extrapulmonary), cannot be interpreted as evidence of a class-specific clinical pattern.

In our case series, the degree of functional impairment did not parallel the CTCAE severity grade, although this observation is limited by missing data. The lowest DLCO of the series (case 2, 17.4% of predicted) was recorded in a patient graded 2 whose oxygen saturation was normal (96%), and three of the four patients with a severe reduction in DLCO (<40%) were graded 2. However, lung function tests could not be obtained in three patients (cases 6, 11 and 12), including the most severe case (case 11, grade 4) and one of the grade 3 patients (case 12). The available functional values therefore reflect predominantly the lower severity grades, and no firm conclusion can be drawn regarding the relationship between functional impairment and CTCAE grade (Table 3).

### 3.2. Radiologic and Bronchoscopy Features

In four of 12 cases, the CT scan showed patchy opacities with mild intensity (ground-glass—GGO) predominantly at the periphery of both lung fields, and these were more consistent with the nonspecific interstitial pneumonia (NSIP)-like pattern. In seven of 12 cases, the opacities were more intense, expressing features of lung consolidation, and in two of them the consolidations were peribronchovascular. These cases were more consistent with an organizing pneumonia (OP) radiologic pattern. Only one case showed diffuse bilateral centrilobular nodules more consistent with a hypersensitivity pneumonitis (HP) radiologic pattern. The CT patterns are centralized in Table 4.

Two patients (cases 9 and 10) developed pneumomediastinum: one at admission, and the other at the 1-month control, a complication not commonly reported in oncologic therapy-induced pneumonitis. In both, it followed a benign course and resolved without specific intervention, even though spontaneous pneumomediastinum has been described as a marker of severe alveolar injury in other interstitial lung diseases.

In three cases CT scan was not performed at the control visit given the clinician’s consideration that the information provided by the clinical picture of the patient and the chest X-ray is sufficient for assessing the improvement.

Only one case (case 11) was not evaluated with a chest CT scan at the moment of pneumonitis diagnosis, as the patient refused to be admitted to the hospital and undergo a CT scan, although the severity was grade 4 and the diagnosis relied only on the clinical data, the nivolumab treatment, and the chest X-ray that showed a patchy interstitial pattern with mild-intensity opacities. The patient received corticotherapy and oxygen therapy at home and improved clinically and radiologically at 1 month, and the CT scan performed at 1 month showed an improved NSIP-like pattern. A comparison was made between chest X-rays performed at the diagnosis and at 3-month intervals and showed complete remission of pneumonitis.

**Figure 2:**
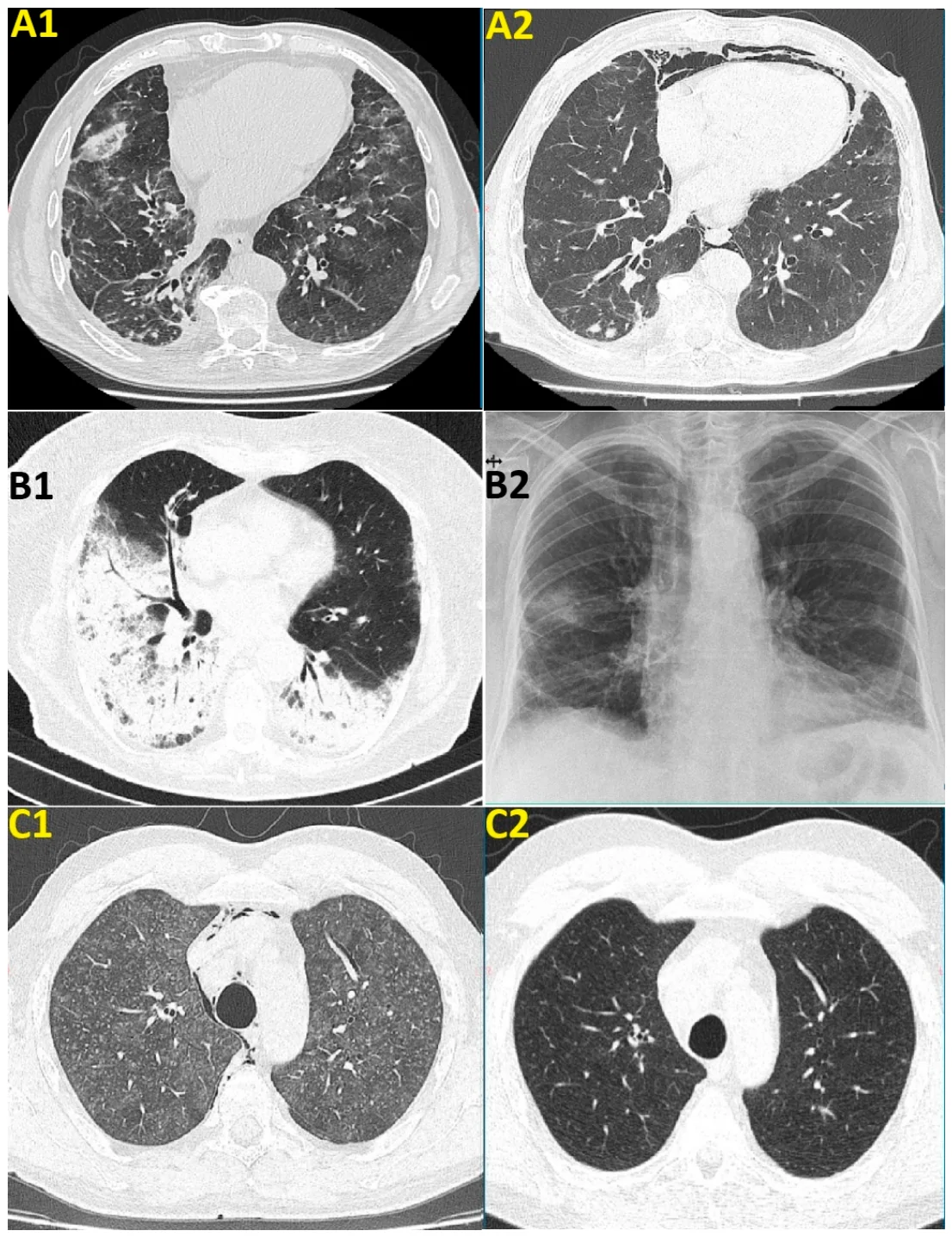
Radiologic patterns of oncologic treatment-induced pneumonitis; (**A1**) case 10 treated with nivolumab—nonspecific interstitial pneumonia (NSIP)-like pattern on thoracic CT; (**A2**) case 10 after 1 month of corticotherapy, with pneumomediastinum; (**B1**) case 6 treated with pembrolizumab—organizing pneumonia (OP) pattern on thoracic CT; (**B2**) case 6’s chest X-ray after 1 month of corticotherapy; (**C1**) case 9 treated with ipilimumab/nivolumab—hypersensitivity pneumonitis (HP) pattern on thoracic CT; (**C2**) case 9 after 1 month of corticotherapy. (See Table 5 and Figure 3 for corticotherapy regimens.).

The three types of radiologic patterns (OP, NSIP and HP) are illustrated in Figure 2.

Bronchoalveolar lavage was performed in eight of the 12 included patients, revealing lymphocyte-predominant cellularity in all cases, with no detection of neoplastic cells. In all cases with late onset (cases 2, 3, 4 and 9), after months of oncologic treatment discontinuation, BAL was performed to exclude alternative diagnoses, but only in four of 8 cases of pneumonitis that occurred during oncologic treatment, BAL was performed. In the other three cases, BAL was not considered necessary based on the high probability of oncologic treatment-associated pneumonitis, and in case 11 the patient declined the procedure, which would in any case have carried an unacceptable risk of further respiratory deterioration given the severe respiratory failure.

### 3.3. Treatment and Outcome

**Figure 3:**
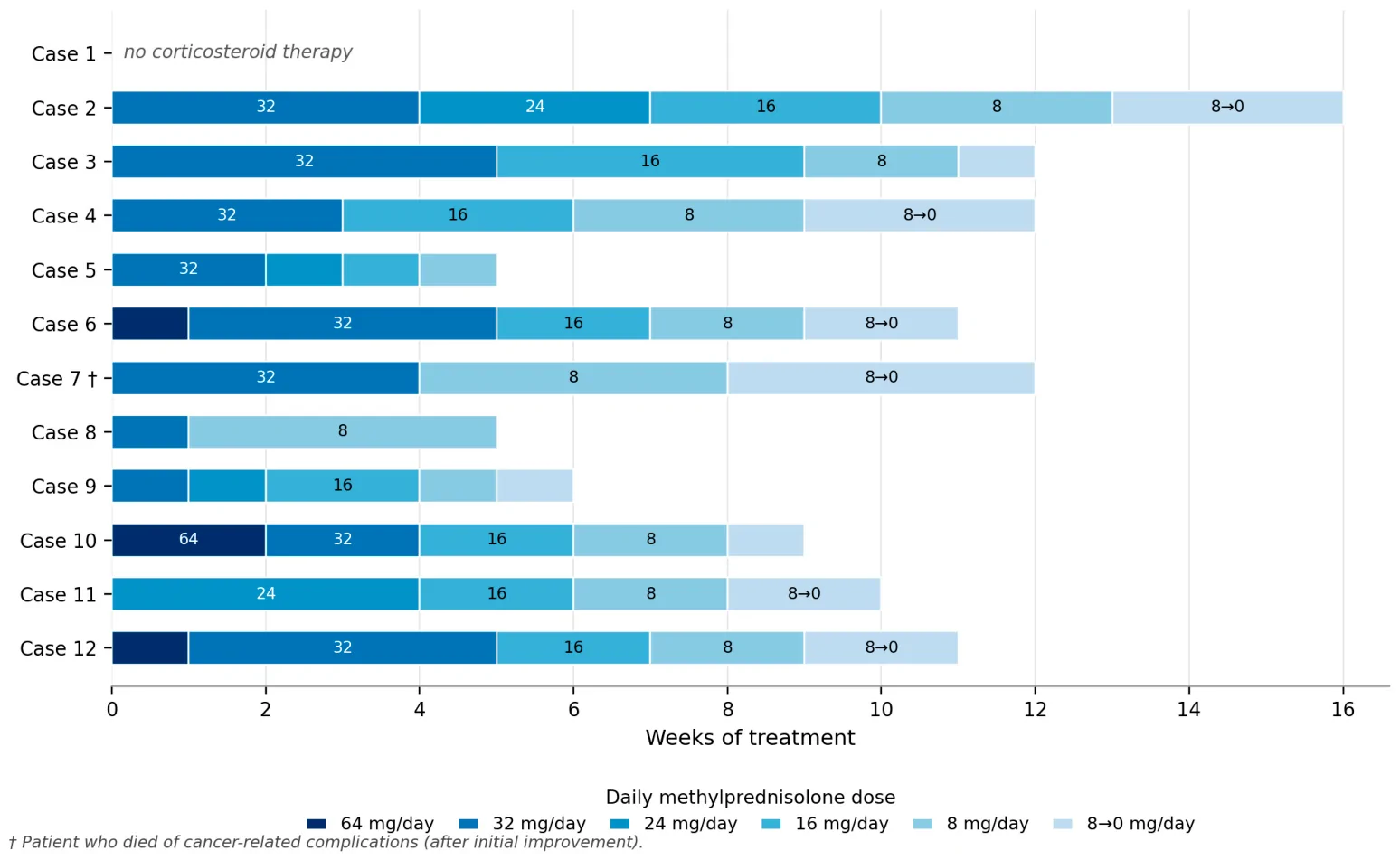
Methylprednisolone tapering regimens in the 11 patients treated with corticosteroids; the width of each colored segment corresponds to the number of weeks spent at the corresponding daily methylprednisolone dose, and the color indicates the dose level (see legend). Case 1 received no corticosteroids and showed spontaneous resolution.

Pneumonitis treatment consisted of oral methylprednisolone (Table 5) starting at moderate doses (ranging from 0.4 to 0.82 mg/kg, with an average of 0.55 mg/kg) in 11 out of 12 patients, with gradual dose tapering throughout 5 to 16 weeks, depending on each patient’s clinical and imaging evolution. The treatment regimens were highly variable and are illustrated in Figure 3. The decision to start with a dose close to the inferior or superior limit of the general recommended interval (equivalent to a prednisone dosage of 0.5 to 1 mg/kg) was made by each clinician based on severity and comorbidities, and the following regimen was also adjusted by each clinician based on treatment response and clinical experience with different types of pneumonitis treatment with corticotherapy.

Immunotherapy or targeted oncological therapy was discontinued in six patients, while in four cases, discontinuation was not necessary as pneumonitis developed after completion of treatment. In two patients, the decision was made to continue the oncologic therapy (in case 1 without and in case 5 in parallel with corticotherapy).

It is noteworthy that the mild symptomatic patient in whom targeted therapy was not discontinued was monitored solely through imaging, without corticosteroid administration, and exhibited spontaneous resolution of the interstitial lung lesions (Case 1).

Symptomatic improvement was achieved in all 12 patients, with resolution of parenchymal lesions in 11 out of 12 cases. The imaging evolution of each patient is illustrated in Appendix A. In Case 7, the oncologic treatment was discontinued, and the patient had experienced improvement after 2 months of corticotherapy. Immunotherapy was reintroduced, in parallel with methylprednisolone administration, and this led to a worsening of pulmonary lesions, despite an improvement in clinical symptoms. Ultimately, the patient died of cancer complications.

## 4. Discussion

Reviewing these 12 cases, a series of discussions are raised. Because our cohort included three distinct drug classes, each aspect is discussed separately for ICI-related, anti-CD20-related, and CDK4/6 inhibitor-related pneumonitis while acknowledging that the small size of the non-ICI groups precludes any direct comparison.

The first aspect is related to diagnosis. In our series, the diagnosis relied on clinical, onco-therapeutic, and radiologic features, with bronchoscopy reserved for selected cases. The recognition of pneumonitis can be difficult, as the clinical presentation and CT appearance can be very similar to other conditions like infection, pulmonary edema, radiation pneumonitis, pulmonary embolism, or tumor progression. All these diagnoses must be ruled out not only at initial diagnosis, but also when the response to treatment is poor, or the symptoms recur.

BAL is generally recommended for grade ≥ 2 to establish the presence of an infection, and it remains unclear whether BAL cell analysis may be useful in the diagnosis of induced ICI pneumonitis. Some real-life studies have shown that induced ICI pneumonitis is associated with BAL lymphocytosis [21,22], and it seems that there are no differences between the chest CT pattern and the BAL cytology [23]. Our findings are consistent with the literature findings. Lavage was performed in eight of 12 patients and showed lymphocyte-predominant cellularity with no malignant cells and negative bacteriology and mycology. Its principal contribution was therefore the exclusion of infection and malignant involvement rather than positive confirmation of pneumonitis.

The diagnostic approach also differed by drug class. In the ICI group, in which pneumonitis is a well-characterized and comparatively frequent adverse event, the clinical–radiologic–contextual triad may usually be sufficient. In the obinutuzumab and abemaciclib groups, the diagnosis rested more heavily on exclusion, since pneumonitis is rarely reported with these agents and the index of suspicion is correspondingly lower. For abemaciclib, the supporting data from clinical trials report interstitial lung disease or pneumonitis of any grade in 3.3% [24], while for obinutuzumab only isolated case reports exist [10,11]. In patients with high-grade severity, in whom bronchoscopy carries additional risk, we consider that clinical, radiological and circumstantial data may reasonably establish a probable diagnosis, with invasive testing reserved for atypical presentations or inadequate corticosteroid response (Figure 1).

The second aspect is related to the CT radiologic patterns. The pattern distribution in our series (7 OP, 4 NSIP-like, 1 HP) is broadly consistent with published cohorts. The OP pattern was the most common form across all the tumor types and ICI regimens [25]. The clinical severity represented by the pneumonitis grades was associated with radiographic patterns so that a pattern-based approach may be useful in assessing severity. The highest CTCAE grades are seen with diffuse alveolar damage and organizing pneumonia patterns and the lowest with NSIP and HP [26]. Although they can be helpful in imagistic descriptions, some are purely descriptive without pathophysiological correspondence and should not be equated with distinct clinicopathological entities sharing the same name.

By drug class, all three obinutuzumab-related cases showed an OP or NSIP-like pattern, in keeping with the two published obinutuzumab reports, both of which described organizing pneumonia [10,11]. The single abemaciclib case showed an OP pattern; the literature on CDK4/6 inhibitors is limited, but a multicenter series of 10 patients with CDK4/6 inhibitor-associated interstitial lung disease found hypersensitivity pneumonitis and NSIP to be the commonest radiological patterns [27].

The relatively late onset in our cohort is consistent with the milder presentations observed. In a cohort of 32 patients, early-onset ICI-induced pneumonitis cases were higher in the CTCAE v5.0 grade, whereas late-onset ICI-induced pneumonitis cases were lower in CTCAE grade and mainly presented with an NSIP-like radiographic pattern. The prognosis of the early-onset pneumonitis was poorer than that of the late-onset pneumonitis group [28].

Also, Huang et al. showed that patients with high-grade pneumonitis were significantly older than those with low-grade disease (65.8 vs. 61.5 years, *p* = 0.03) and had poorer performance status, and older age has been reported as a risk factor for immune-related adverse events in several studies [29]. In our ICI group, however, this association was not reproduced: the four patients graded 3–4 were, on average, younger than those graded 1–2 (a mean of 65 vs 73 years). Given only eight patients in this subgroup, this discrepancy should not be interpreted as contradicting previous reports, but it does indicate that age alone did not determine severity in our cohort.

The third discussion is related to treatment. Currently, there is no standard recommendation on dosages and duration of treatment. In this case series, using highly variable regimens of treatment, the patients had good outcomes, but this descriptive experience cannot establish comparative effectiveness or support a severity-based dosing strategy. Data suggest starting with a dose of 1–2 mg/kg prednisone with 4–6-week taper [18] or longer for patients with grade 3 or 4 disease [30]. The chronic ICI pneumonitis, defined as symptoms lasting > 12 weeks requiring treatment, may require prolonged therapy courses with potential additional immunosuppression and limits the ability to rechallenge with immunotherapy agents [31].

One patient (case 1, abemaciclib, grade 1) improved without corticosteroids and while continuing the targeted therapy, being monitored by imaging alone, with spontaneous resolution of the lung lesions—consistent with guidance that in asymptomatic or minimally symptomatic patients, imaging surveillance without interruption of the anticancer agent may be sufficient.

The fourth discussion concerns whether to maintain or discontinue oncologic treatment. In this subject, there is some controversy, and the decision is a matter of consensus between the pneumologist and oncologist. Guideline recommendations are graded by severity, so patients with grade 1–2 pneumonitis may be considered for rechallenge after complete symptom resolution and completion of the steroid taper, whereas rechallenge is generally discouraged in grade 3–4 disease unless no alternative treatment exists [32,33].

Our experience is consistent. Only two patients continued anticancer therapy, and one deteriorated after reintroduction of immunotherapy. The considerations differ by class: for ICIs, structured guidance on rechallenge exists and was the framework we applied; for obinutuzumab and abemaciclib, no comparable guidance is available, and decisions were necessarily individual. Notably, for CDK4/6 inhibitors, regulatory advice is to evaluate patients with new or worsening respiratory symptoms and to consider dose interruption, modification or discontinuation according to event severity [34]. We therefore consider that the decision should be individualized, based on severity and multidisciplinary discussion, rather than generalized from a small series.

The fifth discussion is related to follow-up. In this case series, three cases were managed successfully without a repeat CT scan, with clinical evaluation and chest radiography being sufficient to document the outcome. Omitting repeat CT in patients with clear clinical improvement, and reserving it for those who do not improve, could reduce costs and patient discomfort. This remains a pragmatic observation rather than a validated strategy: only three of 12 patients were followed in this way, no comparative assessment was possible, and the approach presupposes reliable clinical improvement. Continued vigilance is essential, since recurrence may occur during steroid tapering as well as after rechallenge [33]. The follow-up assessment time interval should be based on severity, and based on this assessment (improved, stationary, worsened), the treatment regimen can be adjusted.

### Limitations

This study has several limitations. First, it is a retrospective, single-center case series based on a small number of patients (n = 12), without a control group, which limits the generalizability of our observations. Second, the cohort is heterogeneous, comprising different tumor types and different classes of anticancer agents (immune checkpoint inhibitors, an anti-CD20 agent, and a CDK4/6 inhibitor), so the groups are not directly comparable and the findings for the individual agents rest on very few cases. Third, the diagnosis of drug-induced pneumonitis was based on the temporal relationship with treatment, the clinical and radiological picture, and the exclusion of alternative causes, without histological confirmation in most patients; only BAL cellularity and bacteriological and mycological examinations were performed. Fourth, pulmonary function tests were not available for all patients, particularly some of the most severely affected, which limits the interpretation of the functional data. Finally, the corticosteroid regimens were heterogeneous and individually tailored, and follow-up was not standardized, so no conclusions can be drawn regarding the comparative effectiveness of different treatment strategies.

Accordingly, our findings should be regarded as descriptive and hypothesis-generating, and they require confirmation in larger, prospective and multicenter studies.

## 5. Conclusions

Immunotherapy and targeted therapy have revolutionized cancer treatment, yielding more favorable outcomes for many patients compared to classic chemotherapy. Although pulmonary toxicity is not a frequent adverse effect, it represents a significant cause of morbidity and mortality in this patient population. The current literature reports a low incidence of immunotherapy- or targeted therapy-induced pneumonitis; however, the trend appears to be increasing due to the growing use of these agents—often in combination—as well as improved recognition of clinical symptoms and interstitial lung abnormalities.

In our series, a probable diagnosis of treatment-induced pneumonitis was reached from clinical, contextual, and radiologic patterns, with BAL performed in eight of the 12 patients. These observations suggest that, in selected patients with a compatible clinical and radiological picture, an accurate diagnosis may be approached with less invasive testing; however, this small, selected sample does not establish that bronchoscopy or CT can generally be avoided. Also, in clinically improving patients, follow-up with clinical assessment and chest radiography appeared feasible—repeat CT was omitted in only three patients—but this should be regarded as a hypothesis to be tested in larger studies.

Timely identification of symptoms, radiological changes, assessment of severity, and prompt start of treatment play a crucial role in the management and prognosis of affected patients. Current guidelines, largely based on expert consensus, recommend discontinuing oncologic treatment in all cases of therapy-induced pneumonitis. However, considering the risk–benefit ratio, our center’s experience suggests that oral corticosteroid therapy in low to moderate doses administered concurrently with immunotherapy or targeted therapy can yield favorable outcomes. Close and continuous monitoring of patients—through regular clinical, functional, and imaging assessments at intervals of one to three months—is essential.

Further studies are needed to develop clear therapeutic protocols, particularly those addressing the continuation, discontinuation, or reintroduction of immunotherapy following the resolution of the acute episode, as well as to identify specific risk factors that may predispose patients to pulmonary toxicity.

## Figures and Tables

**Table 1:** Patient characteristics.

No	Age	S	Cancer	Therapy	Treatment Cycle	Time from Last Cycle to Symptoms of Pneumonitis (Months)
1	50	F	Breast	Abemaciclib	18	1
2	34	F	NHL	Obinutuzumab	6 + 12 maintenance cycles	6
3	47	M	NHL	Obinutuzumab	6 + 8 maintenance cycles	1
4	50	M	NHL	Obinutuzumab	6 + 13 maintenance cycles	6 *
5	74	F	Lung	Pembrolizumab	9	1
6	69	F	Lung	Pembrolizumab	12	<1
7	67	M	Lung	Pembrolizumab	21	<1
8	67	M	Lung	Pembrolizumab	14	<1
9	52	M	Renal	Ipilimumab, Nivolumab	10,21	<1
10	82	M	Lung	Nivolumab	14	12
11	79	F	Lung	Nivolumab	8	1
12	62	F	Lung	Nivolumab	6	<1

Age = age in years; S = sex (M = male; F = female); NHL = non-Hodgkin lymphoma; * symptoms present during treatment but were not investigated for target therapy-induced pneumonitis.

**Table 2:** CTCAE criteria for grading the severity of the pneumonitis.

Grade		Symptoms	Management
1	Mild	Asymptomatic or mild symptoms	Clinical or diagnostic observations only; intervention not indicated.
2	Moderate	Limiting age-appropriate instrumental ADL.	Minimal, local, or noninvasive intervention indicated.
3	Severe	Severe or medically significant, but not immediately life-threatening; disabling; limiting self-care ADL.	Hospitalization or prolongation of hospitalization indicated.
4	Life-threatening	Life-threatening consequences	Urgent intervention indicated.
5	Death		Death related to the adverse event.

ADL = Activities of Daily Living.

**Table 3:** Onset time in months from start of oncologic treatment, symptoms, severity and lung function tests data.

No	Onset (Months)	Symptoms				Severity Grade	SpO_2_ (%)	FVC (%)	DLCO (%)
		Dyspnea	Cough	Fever	Others				
1	19	No	Yes	No	No	1	99	77	54
2	24	Yes	No	No	No	2	96	59.4	17.4
3	24	Yes	Yes	No	LA	2	99	NA *	68
4	48	No	Yes	No	CP	2	98	61	65
5	12	Yes	No	No	No	2	95	75.8	50.5
6	18	Yes	Yes	No	No	2	98	NP	NP
7	16	Yes	No	No	No	2	99	64.3	38.6
8	10	Yes	No	Yes	No	3	88	84.7	NP
9	24	Yes	No	No	No	3	92	68.2	21.9
10	24	Yes	No	No	No	2	98	46.2	29.6
11	8	Yes	Yes	No	No	4	70	NP	NP
12	6	Yes	Yes	No	No	3	88	NP	NP

LA = loss of appetite; CP = chest pain; SpO_2_ = oxygen saturation of hemoglobin; DLCO = lung diffusion of carbon monoxide in percentage of predicted value; FVC = forced vital capacity in percentage of predicted value; NA = not available; NP = not performed. * normal values.

**Table 4:** Radiographic findings.

Case	Radiographic Pattern	CT Findings	BAL
1	OP	Bilateral subpleural consolidations	Yes
2	OP	Bilateral peripheral consolidations, left pleural effusion	Yes
3	OP	Bilateral multifocal and migratory GGOs	Yes
4	NSIP	Bilateral GGOs	Yes
5	OP	Bilateral peribronchovascular consolidations	Yes
6	OP	Bilateral multifocal consolidations and GGOs	No
7	OP	Bilateral large lung consolidations	Yes
8	OP	Bilateral peribronchovascular consolidations	Yes
9	HP	Diffuse bilateral centrilobular ground-glass nodules, mosaic attenuation caused by the air trapping; pneumomediastinum	Yes
10	NSIP	Bilateral predominantly peripheral reticular opacities and GGOs;	No
11	NSIP	Bilateral GGOs	No
12	NSIP	Bilateral predominantly peripheral reticular opacities and GGOs	No

OP = organizing pneumonia; HP = hypersensitivity pneumonitis; NSIP = nonspecific interstitial pneumonia; GGOs = ground-glass opacities; BAL = bronchoalveolar lavage made/not made.

**Table 5:** Treatment, decision to stop oncologic treatment and outcome.

No	Weight	Dose	Oral CS	Weeks of Methylprednisolone Dose (mg/day)						Hold	Outcome
	kg	mg/kg	(Weeks)	64	32	24	16	8	8/0		
1	64	N/A	0	0	0	0	0	0	0	No ++	Improved
2	70	0.46	16	0	4	3	3	3	3	No need	Improved
3	78	0.41	12	0	5	0	4	2	1	No need	Improved
4	70	0.46	12	0	3	0	3	3	3	No need	Improved
5	65	0.49	5	0	2	1	1	1	0	No ++	Improved
6	78	0.82	11	1	4	0	2	2	2	Yes	Improved
7	79	0.41	12	0	4	0	0	4	4	Yes +	Death *
8	80	0.40	5	0	1	0	0	4	0	Yes	Improved
9	75	0.43	6	0	1	1	2	1	1	No need	Improved
10	82	0.78	9	2	2	0	2	2	1	Yes	Improved
11	42	0.57	12	0	0	4	2	2	2	Yes	Improved
12	78	0.82	11	1	4	0	2	2	2	Yes	Improved

CS = corticosteroid (duration in weeks); Hold = hold oncologic targeted treatment. + Initially stopped and after improvement, received immunotherapy and oral corticosteroid simultaneously. ++ Ongoing treatment. * Initial improvement; death by cancer complications.

**Figure A1:**
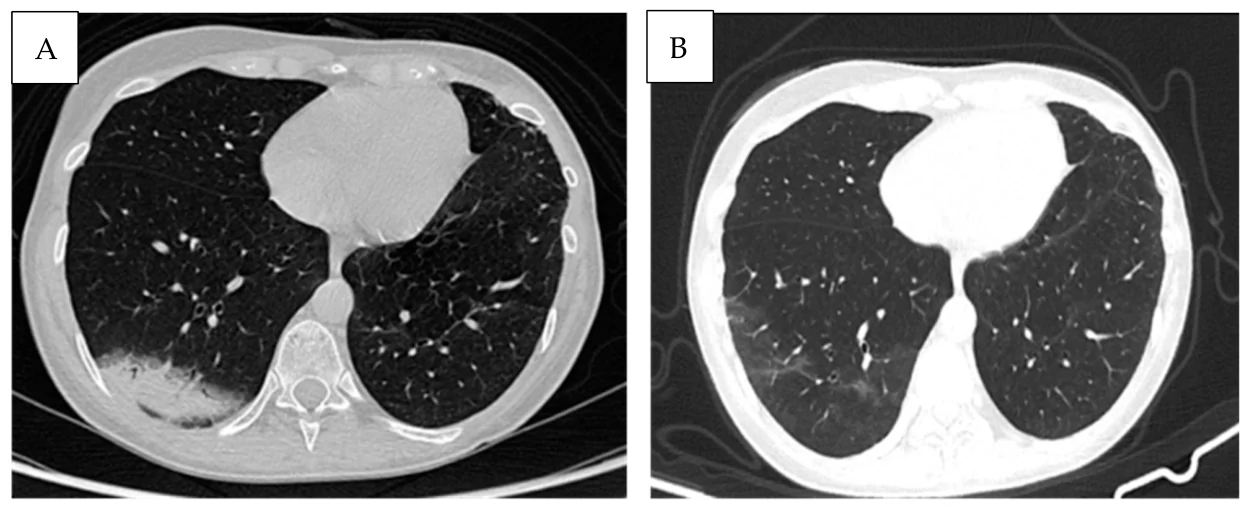
Case 1: Patient with breast cancer treated with abemaciclib presented with an organizing pneumonia (OP) pattern on chest CT (**A**). The lesions resolved after 3 months without corticosteroid therapy or interruption of the oncologic treatment (**B**).

**Figure A2:**
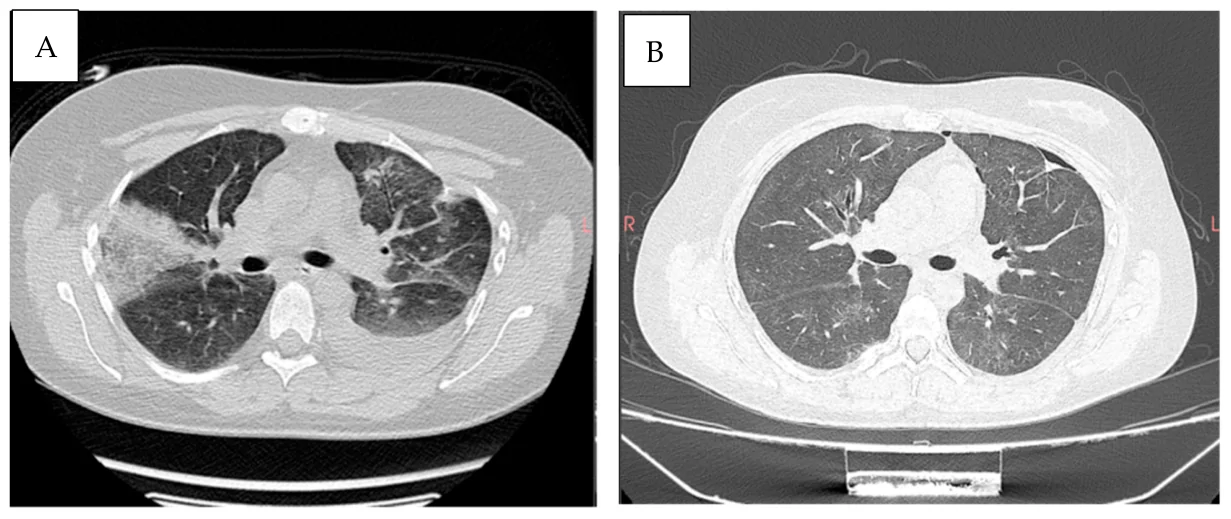
Case 2: A patient with non-Hodgkin lymphoma treated with obinutuzumab, presenting with an organizing pneumonia (OP) pattern on CT and a left pleural effusion (**A**), with improvement of the pulmonary consolidations and resolution of the pleural effusion after 2 weeks of oral corticosteroid therapy (**B**).

**Figure A3:**
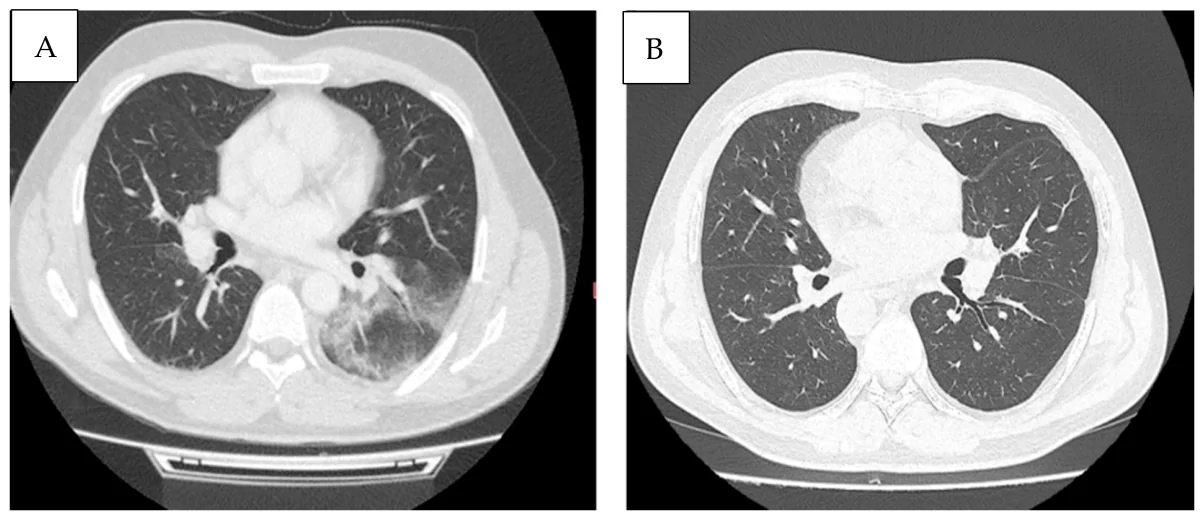
Case 3: Patient with non-Hodgkin lymphoma treated with obinutuzumab presented with an organizing pneumonia (OP) pattern on chest CT (**A**). The lesions resolved after 2 months with corticosteroid therapy (**B**).

**Figure A4:**
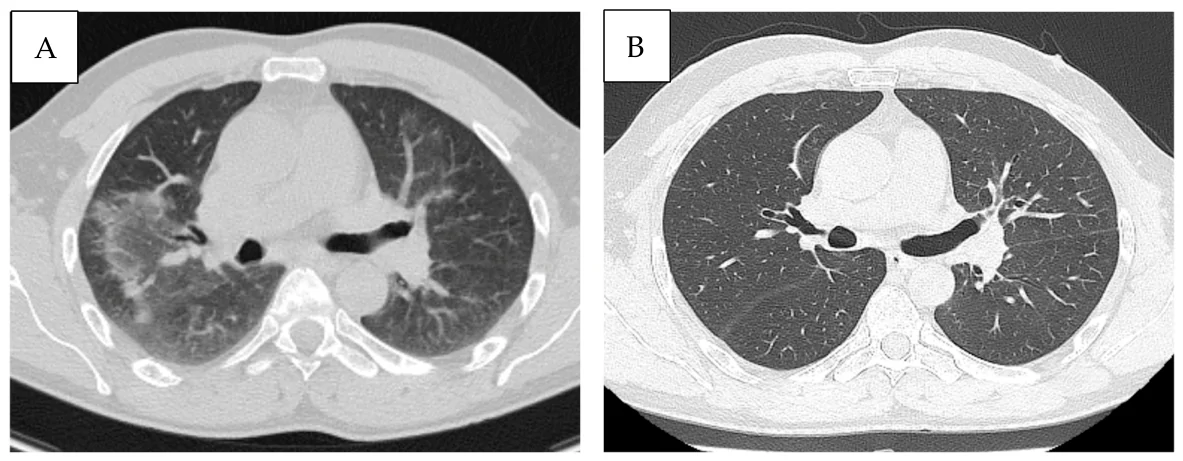
Case 4: Patient with non-Hodgkin lymphoma treated with obinutuzumab presented with NSIP-like pattern on chest CT (**A**). Complete resolution of ground-glass opacities after 3 months of oral corticosteroid therapy (**B**).

**Figure A5:**
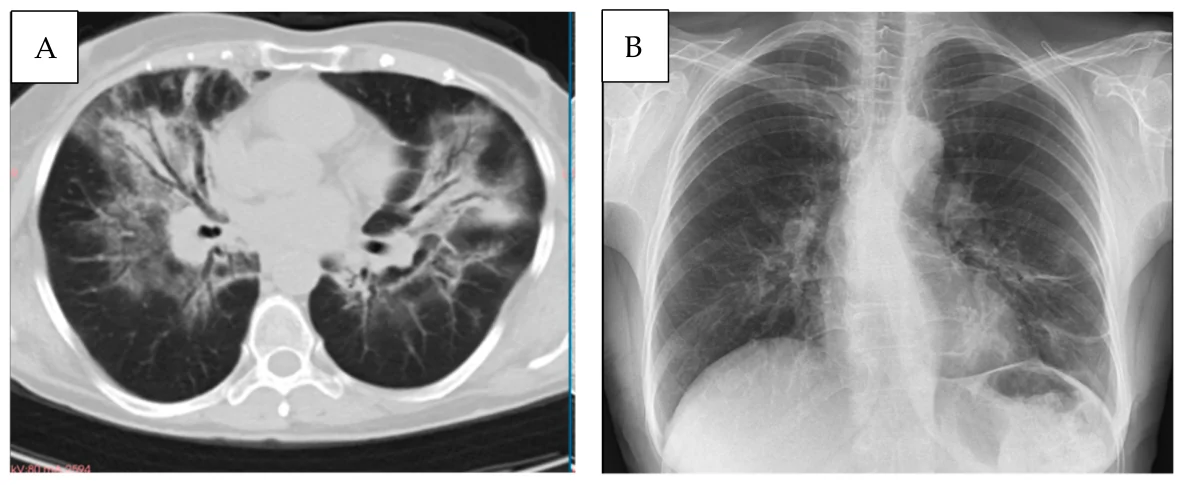
Case 5: Patient with lung cancer treated with pembrolizumab presented with an organizing pneumonia (OP) pattern on chest CT (**A**). Improvement of the pulmonary perihilar lesions on chest X-ray after 1 month with corticosteroid therapy and no interruption of the oncologic treatment (**B**).

**Figure A6:**
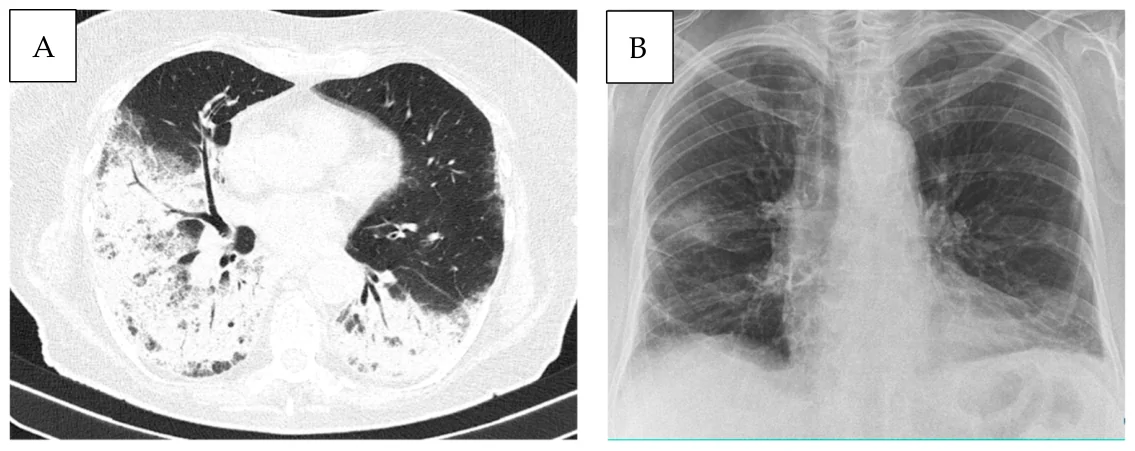
Case 6: Patient with lung cancer treated with pembrolizumab presented with an organizing pneumonia (OP) pattern on chest CT (**A**), with improvement of the lesions on the chest X-ray after one month of corticosteroid therapy and interruption of the oncologic treatment (**B**).

**Figure A7:**
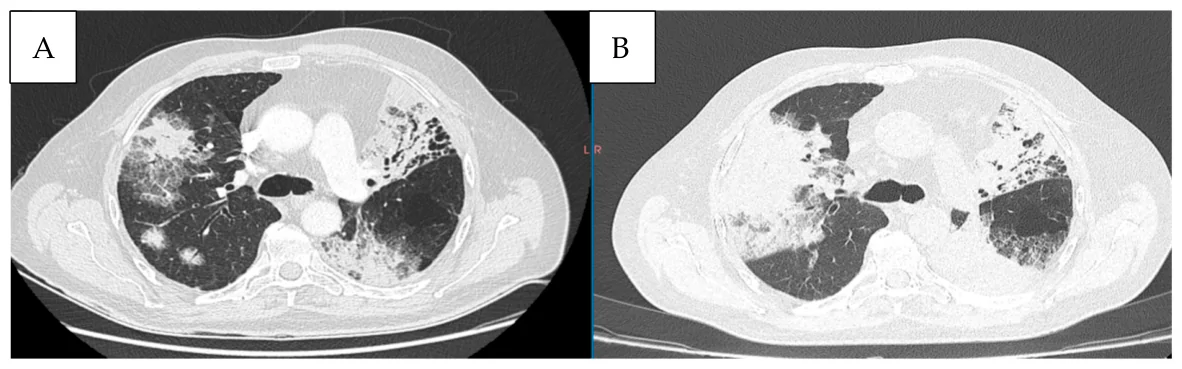
Case 7: Patient with lung cancer treated with pembrolizumab presented with an organizing pneumonia (OP) pattern on chest CT (**A**), with progression of the lesions after reintroduction of the immunotherapy in parallel with corticotherapy (**B**).

**Figure A8:**
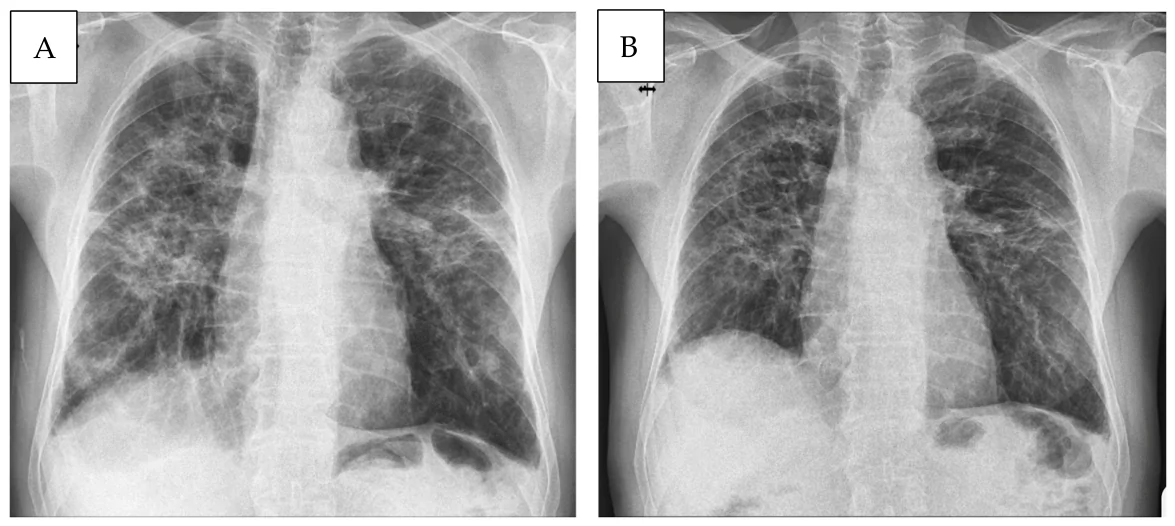
Case 8: Patient with lung cancer treated with pembrolizumab presented with bilateral reticular and alveolar opacities on chest X-ray (**A**). Improvement of the lesions on the X-ray after one month of corticosteroid therapy and interruption of the oncologic treatment (**B**).

**Figure A9:**
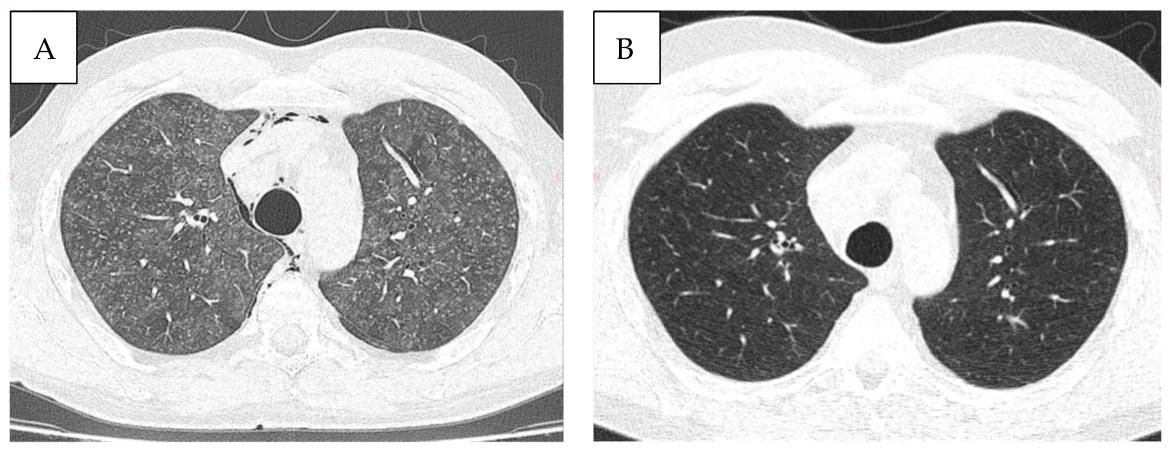
Case 9: Patient with renal cancer treated with ipilimumab/nivolumab presented with an HP pattern on chest CT (**A**). The lesions resolved after one month of corticosteroid therapy (**B**).

**Figure A10:**
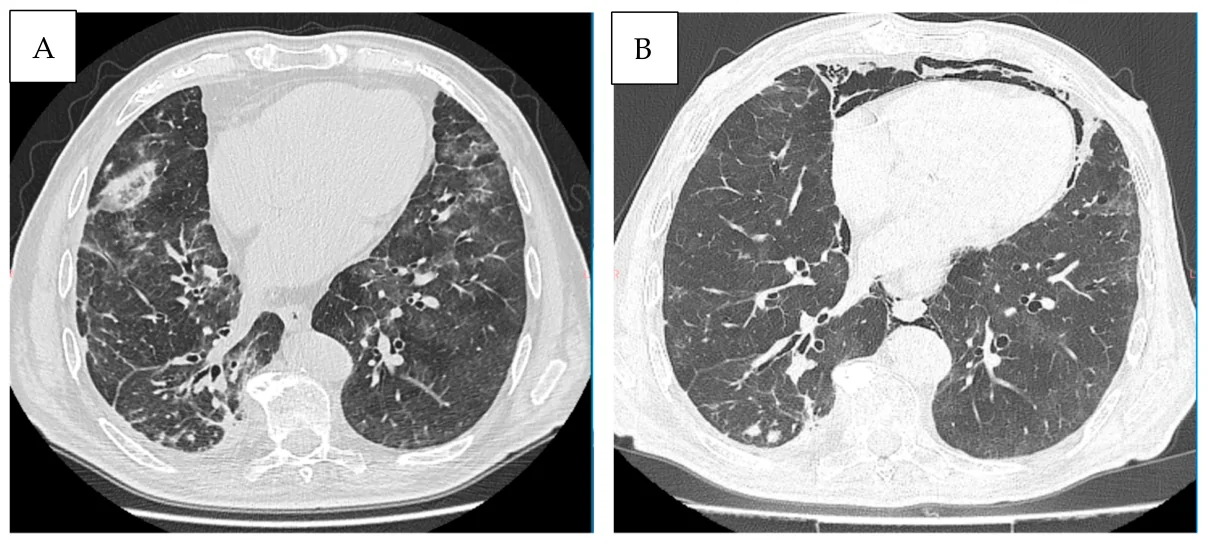
Case 10: Patient with lung cancer treated with nivolumab presented with an NSIP-like pattern on chest CT (**A**). Improvement of the lesions on CT after one month of corticosteroid therapy and interruption of the oncologic treatment (**B**).

**Figure A11:**
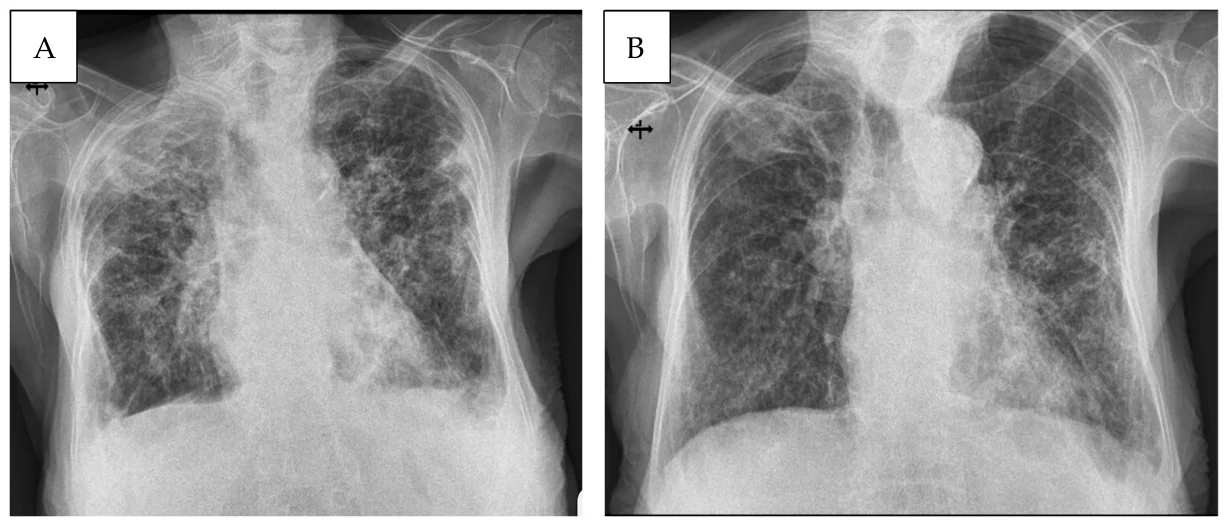
Case 11: Patient with lung cancer treated with nivolumab presented with bilateral reticular opacities on chest X-ray (**A**). Improvement of the lesions on the X-ray after one month of corticosteroid therapy and interruption of the oncologic treatment (**B**).

**Figure A12:**
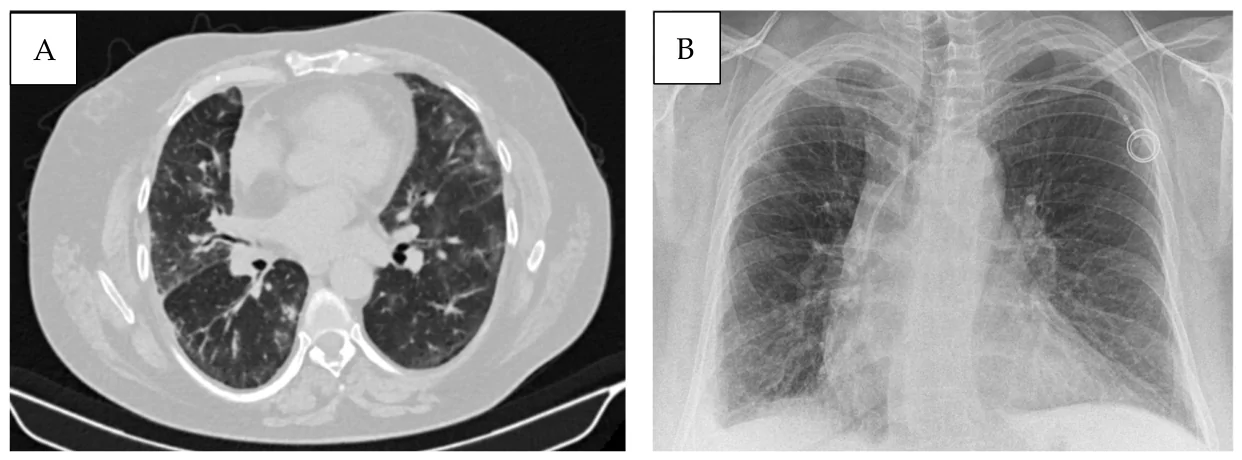
Case 12: Patient with lung cancer treated with nivolumab presented with NSIP-like pattern on chest CT (**A**). The X-ray showed no new lesions, only small pleural effusion on the right side after one month of corticosteroid therapy and interruption of the oncological treatment (**B**).

## Data Availability

The original contributions presented in this study are included in the article. The individual patient data supporting the reported results are not publicly available due to privacy and ethical restrictions regarding patient confidentiality, but anonymized data can be made available on reasonable request from the corresponding authors.

## Abbreviations

The following abbreviations are used in this manuscript:

ARDS	Acute respiratory distress syndrome
BAL	Bronchoalveolar lavage
BL	Bronchiolitis
CD20	Cluster of differentiation 20
CDK 4/6	Cyclin-dependent kinase 4 and 6
CT	Computed tomography
CTCAE	Common Terminology Criteria for Adverse Events
DAD	Diffuse alveolar damage
DAH	Diffuse alveolar hemorrhage
FDA	Food and Drug Administration
HP	Hypersensitivity pneumonitis
ICI	Immune checkpoint inhibitor
HRCT	High-resolution computed tomography
NSIP	Nonspecific interstitial pneumonia
OP	Organizing pneumonia
PaO_2_	Arterial partial pressure of oxygen
RRP	Radiation recall pneumonitis
SARS-CoV2	Severe acute respiratory syndrome coronavirus 2
SLR	Sarcoid-like reactions
SpO_2_	Peripheral oxygen saturation of hemoglobin
